# Open Science and Research Reproducibility

**DOI:** 10.3332/ecancer.2016.ed56

**Published:** 2016-06-07

**Authors:** Marcus Munafò

**Affiliations:** 1 MRC Integrative Epidemiology Unit at the University of Bristol, Oakfield House, Oakfield Grove, Bristol BS8 2BN, UK; 2 UK Centre for Tobacco and Alcohol Studies, School of Experimental Psychology, University of Bristol, Bristol, UK

## Abstract

Many scientists, journals and funders are concerned about the low reproducibility of many scientific findings. One approach that may serve to improve the reliability and robustness of research is open science. Here I argue that the process of pre-registering study protocols, sharing study materials and data, and posting preprints of manuscripts may serve to improve quality control procedures at every stage of the research pipeline, and in turn improve the reproducibility of published work.

Two issues that are prominent in science at present are reproducibility and open science. At first glance, these may seem quite unrelated – the former relates to ongoing concerns that many academic research findings may be incorrect [1], while the latter reflects the movement to encourage openness in science, through open access publication, open data archiving, and so on [2]. My argument is that the open science practices could in fact serve to improve the reproducibility of published work. Why do I think this?

It has been suggested that the incentive structures within which scientists operate may not promote behavior that maximizes the value of scientific research. Novel, groundbreaking results are more likely to be published in high-profile journals, and publication in these journals is more likely to lead to career advancement [3]. This may lead scientists to focus their resources on exploratory work, at the expense of confirmatory work. Researchers may also divide their resources into multiple projects, in the hope that this will increase the chances of one generating a novel result. This will mean that many studies may be underpowered, increasing the probability that a statistically significant finding is in fact a false positive [4]. Researchers may also exploit analytical flexibility to obtain a statistically significant finding (a process known as ‘p-hacking’), and then present these results as if they were anticipated *a priori* (also known as HARKing – Hypothesizing After the Results are Known). When results are perceived to be unexciting (i.e., null), researchers may be less inclined to devote time and effort to writing these up for publication [5], leading to widespread publication bias. The focus on positive results may also be reflected in citation biases, whereby those null results that *are* published received proportionately fewer citations than positive results on the same topic [6]. The consequence of this is a literature that is heavily skewed towards discovery rather than replication, and looks very unlike the totality of the activity that generated it.

There is clearly concern that these issues are undermining the value of science – in the UK the Academy of Medical Sciences recently convened a meeting, jointly with a number of other funders, to explore these issues, while in the US the National Institutes of Health has an ongoing initiative to improve research reproducibility [7]. How can these issues be addressed? There is scope for change at both the level of the wider incentive structures within which scientists operate, and at the level of the day-to-day activity of individual researchers and research groups. Funders and journals, for example, can mandate change – when the National Heart, Lung and Blood Institute required the pre-registration of primary outcomes in clinical trials that they funded, the proportion of studies reporting a benefit of the intervention over the comparator declined dramatically, relative to studies published before this requirement [8]. A number of exciting initiatives are emerging. For example, several journals now offer a Registered Reports format [9], whereby acceptance in principle is offered on the basis of a detailed study protocol, prior to any data collection taking place, on the basis that the results of a well-designed study should always be informative and valuable. In principle, funders and journals could work together, offering acceptance in principle together with funding to conduct the research.

What can individual researchers and groups do? My group, the Tobacco and Alcohol Research Group, part of the MRC Integrative Epidemiology Unit at the University of Bristol and the UK Centre for Tobacco Studies, has been gradually moving towards an open science model over the past few years. We now routinely pre-register studies that involved collection of new data on the Open Science Framework, and share the resulting data via the University of Bristol Research Data Repository (other non-institutional repositories also exist, such as the Open Science Framework and Mendeley Data). This has been a gradual process that has entailed updating a number of our internal procedures [2]. Moreover, it is an ongoing process – we are currently exploring sharing our study materials via platforms such as the Open Science Framework, and posting preprints of our work ahead of publication (for example on bioRxiv). Other enhancements to these processes are possible – for example, the use of research resource identifiers in subsequent publications should allow other researchers to identify exactly the materials used. In our opinion the benefits outweigh this modest effort. Not only is transparency and openness a valuable end in itself (particularly when research is publicly-funded), but it also serves as a quality control process. When researchers know that their study protocol or their data will be available for public scrutiny, they will be inclined to triple-check where previously they would have double-checked. Science is a human endeavour, and honest error is inevitable (witness typographical errors in published manuscripts, despite multiple rounds of peer review, proof reading, and so on). We need to embed processes in our work that help catch these errors, and foster an open culture whereby self-reporting of errors is encouraged.

## Conclusion

Biomedical science is not in crisis, but recent debates around reproducibility offer an opportunity – to reflect on how we conduct science, consider whether our procedures and the incentive structures within we work are optimal, and subject new approaches to empirical scrutiny, in order to establish whether they do improve the quality of scientific outputs. We are in the midst of a large natural experiment whereby various frameworks and platforms are on offer. The open science movement lies at the heart of this, and the experience of my group is that openness offers a number of benefits. My hope is that others will take part in this exciting natural experiment.

## References

[1] Ioannidis JP (2005). Why most published research findings are false. PLoS Med.

[2] Attwood AS, Munafo MR (2016). Navigating an open road. J Clin Epidemiol.

[3] van Dijk D, Manor O, Carey LB (2014). Publication metrics and success on the academic job market. Curr Biol.

[4] Button KS, Ioannidis JP, Mokrysz C (2013). Power failure: why small sample size undermines the reliability of neuroscience. Nat Rev Neurosci.

[5] Franco A, Malhotra N, Simonovits G (2014). Social science. Publication bias in the social sciences: unlocking the file drawer. Science.

[6] Bastiaansen JA, de Vries YA, Munafo MR (2015). Citation distortions in the literature on the serotonin-transporter-linked polymorphic region and amygdala activation. Biol Psychiatry.

[7] Collins FS, Tabak LA (2014). Policy: NIH plans to enhance reproducibility. Nature.

[8] Kaplan RM, Irvin VL (2015). Likelihood of null effects of large NHLBI clinical trials has increased over time. PLoS One.

[9] Chambers CD (2013). Registered reports: a new publishing initiative at cortex. Cortex.

